# Supplementary figures and images for: Spatial and social determinants of the 1857 yellow fever epidemic in Lisbon
Source: PLoS Negl Trop Dis. 2026 Mar 9;20(3):e0014059. doi: 10.1371/journal.pntd.0014059 (PMC12998949; doi:10.1371/journal.pntd.0014059)

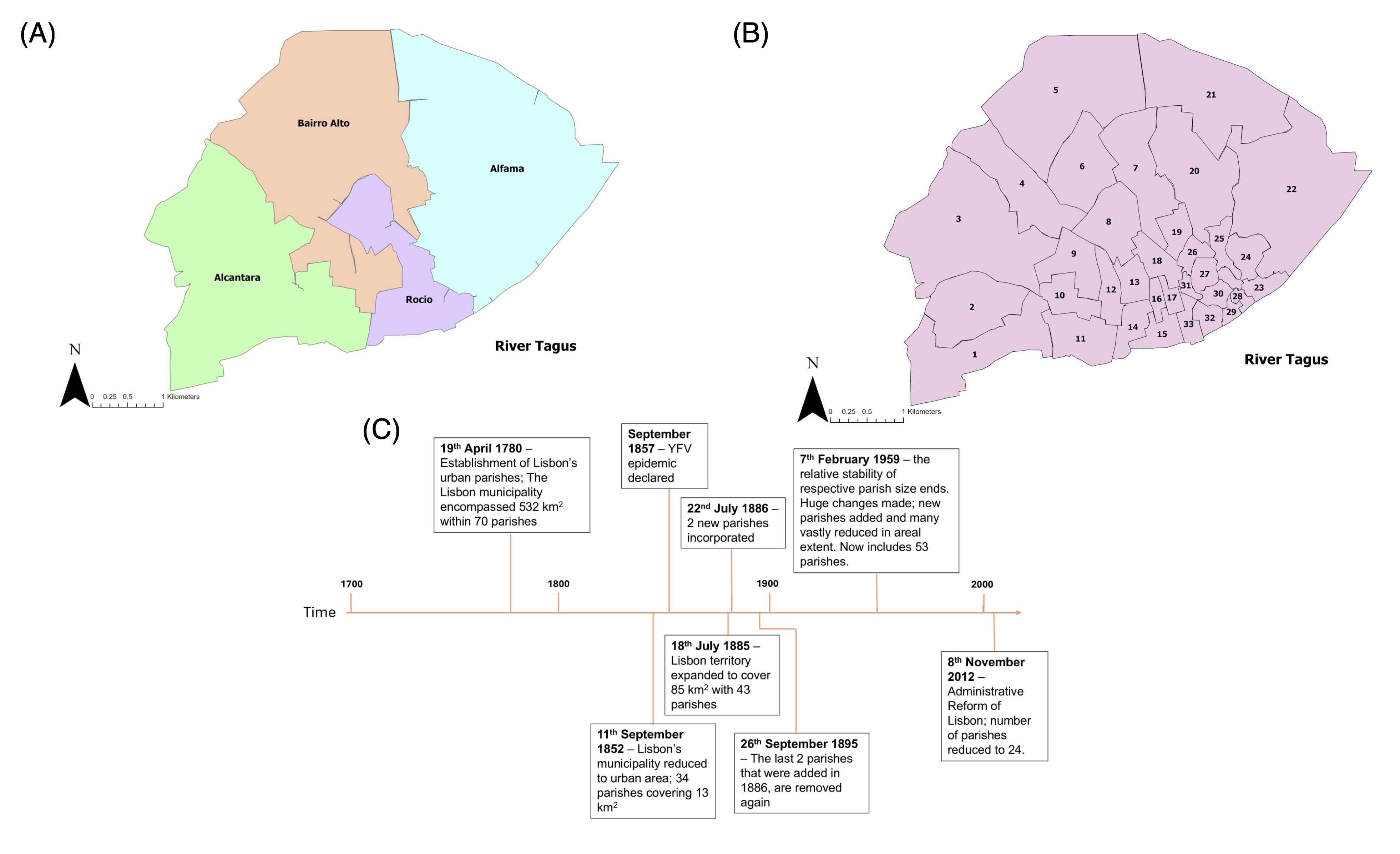

Supplement: S1 Fig — (A) Digitised mid-nineteenth century Lisbon neighbourhood boundaries. (B) Digitised mid-nineteenth century Lisbon parish boundaries located on the bank of the river Tagus. Each parish is numbered – S1 Table provides information on the name of the parish that each number corresponds to. Map scales are 1:27,500. Digital neighbourhood and parish boundaries created by the authors, based on the information in ATLAS Cartografía Histórica [52]. (C) A timeline of the changing municipal organisation of Lisbon [40]. (TIFF) [file pntd.0014059.s004.tiff]

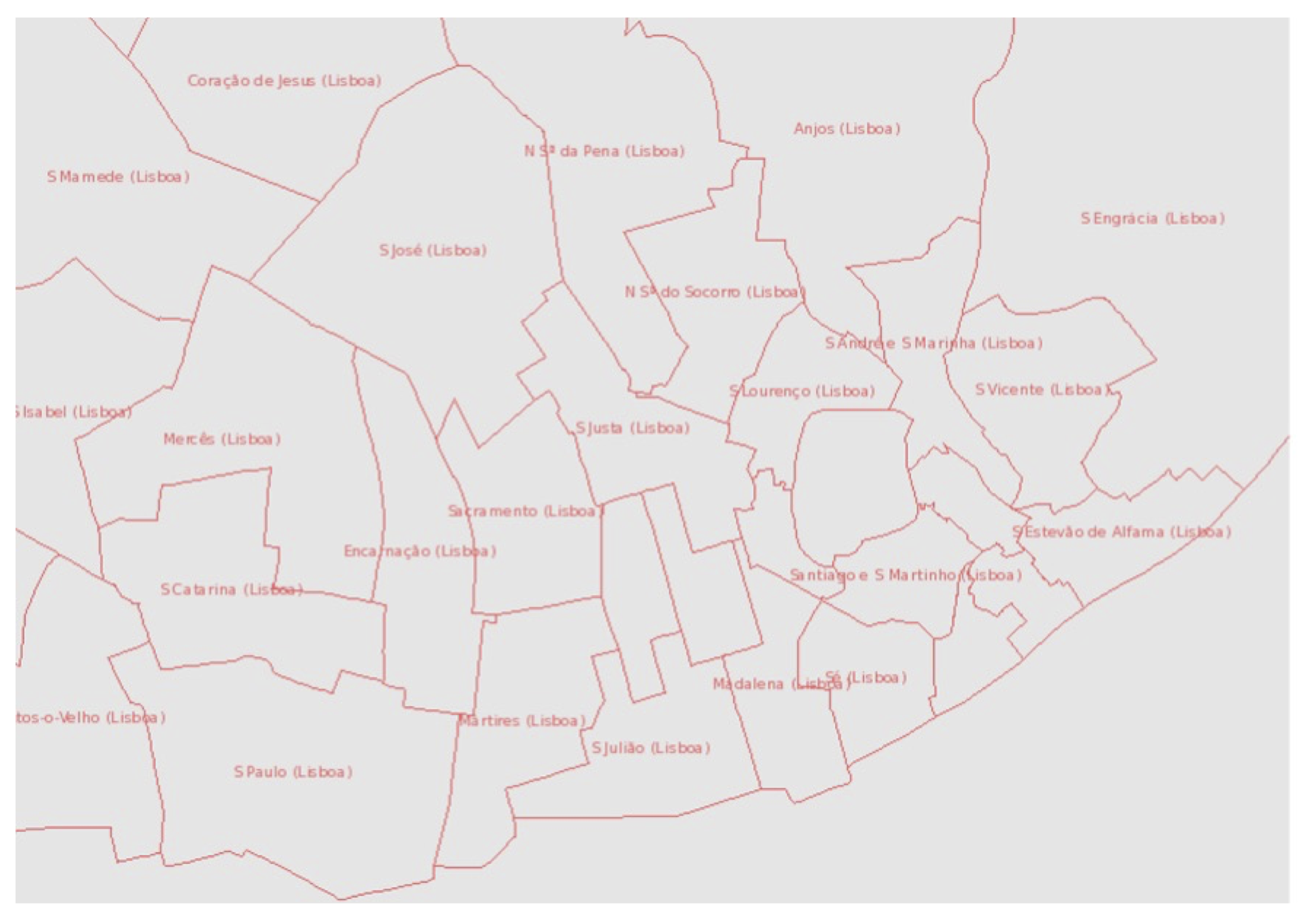

Supplement: S2 Fig — (TIFF) [file pntd.0014059.s005.tiff]

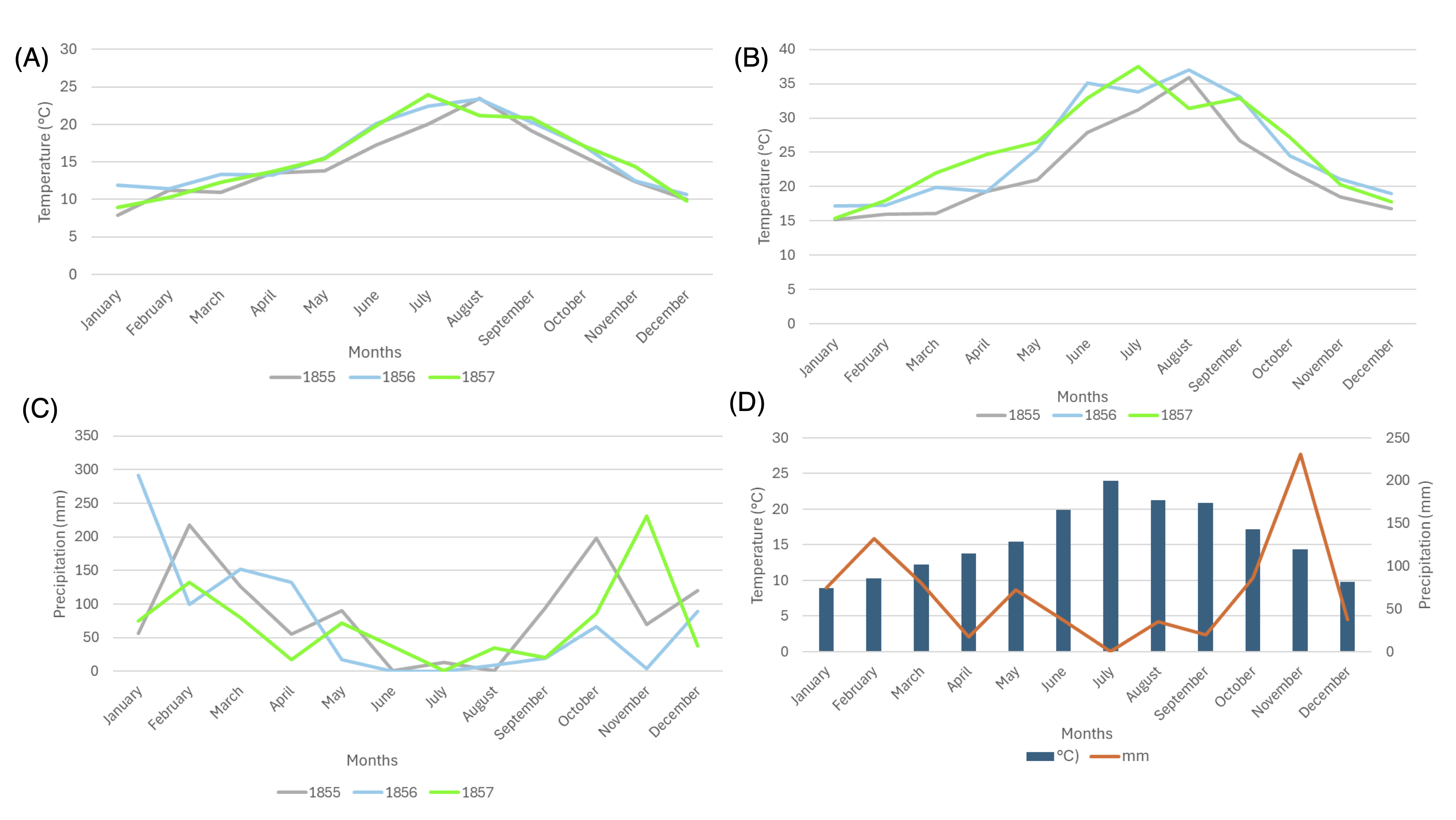

Supplement: S3 Fig — (A) Three-year monthly temperature mean comparison (B) Three-year monthly temperature maxima comparison (C) Three-year monthly total precipitation in Lisbon comparison and (D) Relationship between monthly temperature means and total precipitation throughout 1857. Source: data from Royal Observatory, in Lyons [42]. (TIFF) [file pntd.0014059.s006.tiff]

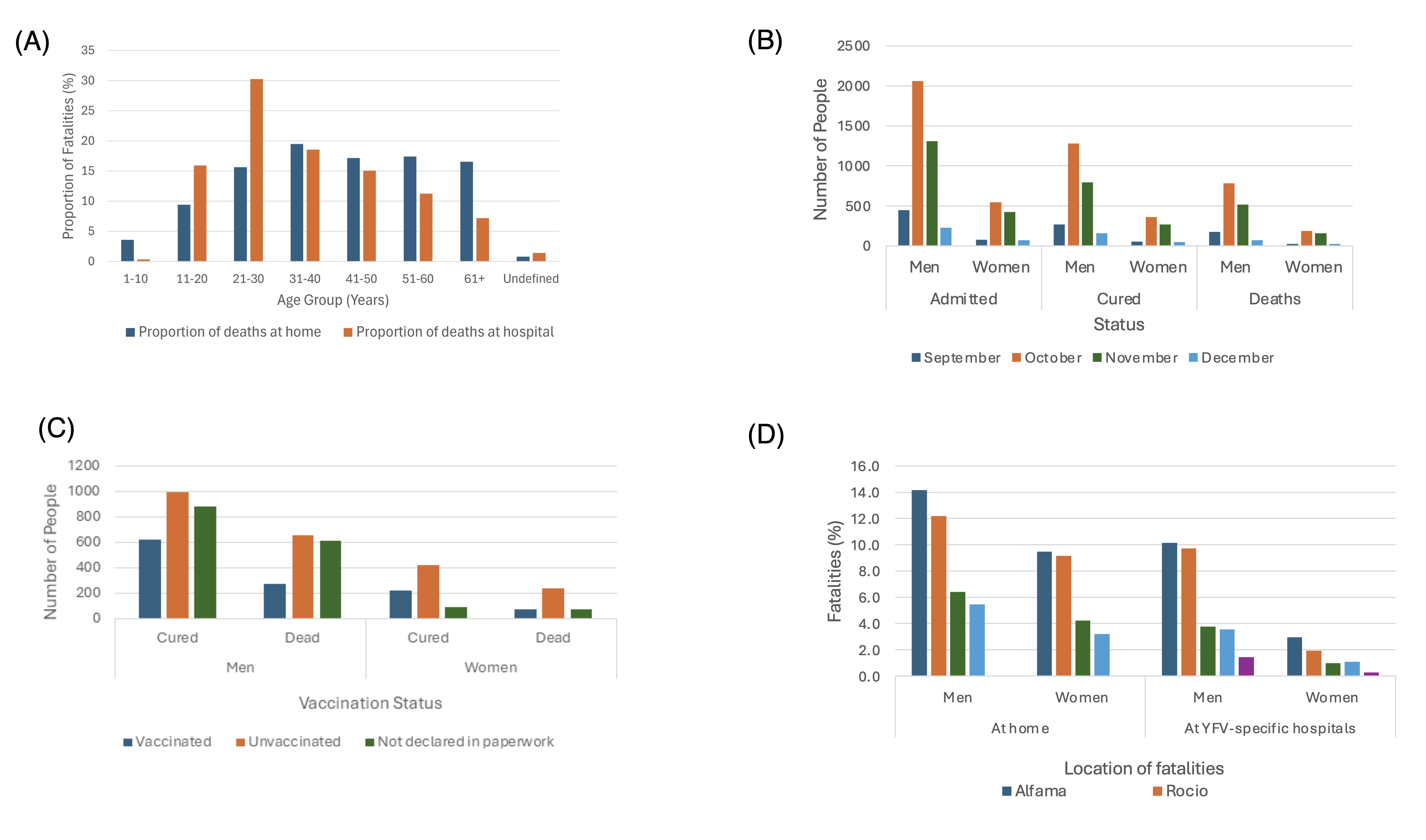

Supplement: S4 Fig — (A) Total deaths by age group at either home or YFV-specific hospital, as a proportion of total deaths in these locations. Age-related information was unavailable (or ‘undefined’) for 0.8% of fatalities at home and 1.4% of fatalities at YFV-specific hospitals. (B) A comparison of admitted, cured, and dead at hospital by sex, with a temporal dimension. (C) The effectiveness of the vaccine, by sex. (D) The distribution of fatalities at home or YFV-specific hospital, by sex and neighbourhood. (TIFF) [file pntd.0014059.s007.tiff]
