# Supplementary material for: Spatial and social determinants of the 1857 yellow fever epidemic in Lisbon
Source: PLoS Negl Trop Dis. 2026 Mar 9;20(3):e0014059. doi: 10.1371/journal.pntd.0014059 (PMC12998949; doi:10.1371/journal.pntd.0014059)
Supplement: Attachment — Submitted filename: 1857 YFV PLoS Review Comments.docx [file pntd.0014059.s009.docx]

**To the Editorial Team:**

We thank all reviewers for their suggestions and comments on the manuscript. We have responded to each comment and suggestion point-by-point below and have made revisions to the manuscript accordingly.

Regarding copyright, we have now added a direct link to the base layer of each map and the terms and conditions of its licensing.

Regarding licensing, we have made changes to the base layer of Fig 5, which now comes from a Digital Terrain Model (DTM) derived from the Copernicus DEM, which we used in our original figure. This represents a “bare-earth” elevation by removing the height contributions of buildings and forest canopy, which reduces distortion from Lisbon’s modern skyline and therefore provides a topography that is more representative of the city’s historical terrain. The DTM uses a creative commons license. This does not change the results that are currently presented.

**Journal Requirements**

If the reviewer comments include a recommendation to cite specific previously published works, please review and evaluate these publications to determine whether they are relevant and should be cited. There is no requirement to cite these works unless the editor has indicated otherwise.

**Journal Requirements, A:** 1) Please ensure that the CRediT author contributions listed for every co-author are completed accurately and in full.
At this stage, the following Authors/Authors require contributions: Isaac Bates, Sabrina Li, Kris Parag, Katy Gaythorpe, Ana Abecasis, Matthew Smallman-Raynor, and Nuno Faria. Please ensure that the full contributions of each author are acknowledged in the "Add/Edit/Remove Authors" section of our submission form.
The list of CRediT author contributions may be found here: <https://journals.plos.org/plosntds/s/authorship#loc-author-contributions>

**Response to Journal Requirements, A:** The CRediT author contributions have been completed accurately and full.

**Journal Requirements, B:** 2) Please provide an Author Summary. This should appear in your manuscript between the Abstract (if applicable) and the Introduction, and should be 150-200 words long. The aim should be to make your findings accessible to a wide audience that includes both scientists and non-scientists. Sample summaries can be found on our website under Submission Guidelines: <https://journals.plos.org/plosntds/s/submission-guidelines#loc-parts-of-a-submission>

**Response to Journal Requirements, B:** We have now written an Author Summary between the Abstract and Introduction. This provides an overview of the wider problem and how our research addresses it. The Author Summary is catered for technical and non-technical audiences alike.

**Journal Requirements, C:** 3) We do not publish any copyright or trademark symbols that usually accompany proprietary names, eg ©,  ®, or TM  (e.g. next to drug or reagent names). Therefore please remove all instances of trademark/copyright symbols throughout the text, including:
- © on page: 16.

**Response to Journal Requirements, C:** The © symbol has been removed from Figure 5. We have changed the source of elevation data to Neal and Hawker (2023), which is licensed under CC BY-NC-SA 4.0.

**Journal Requirements, D:** 4) Please upload all main figures as separate Figure files in .tif or .eps format. For more information about how to convert and format your figure files please see our guidelines: 
<https://journals.plos.org/plosntds/s/figures>

**Response to Journal Requirements, D:** All main figures are uploaded as separate .tif files.

**Journal Requirements, E:** 5) We have noticed that you have uploaded Supporting Information files, but you have not included a list of legends. Please add a full list of legends for your Supporting Information files after the references list.

**Response to Journal Requirements, E:** We have now included a list of legends for the Supporting Information files below the bibliography in the manuscript.

**Journal Requirements, F:** 6) Some material included in your submission may be copyrighted. According to PLOSu2019s copyright policy, authors who use figures or other material (e.g., graphics, clipart, maps) from another author or copyright holder must demonstrate or obtain permission to publish this material under the Creative Commons Attribution 4.0 International (CC BY 4.0) License used by PLOS journals. Please closely review the details of PLOSu2019s copyright requirements here: PLOS Licenses and Copyright. If you need to request permissions from a copyright holder, you may use PLOS's Copyright Content Permission form.
Please respond directly to this email and provide any known details concerning your material's license terms and permissions required for reuse, even if you have not yet obtained copyright permissions or are unsure of your material's copyright compatibility. Once you have responded and addressed all other outstanding technical requirements, you may resubmit your manuscript within Editorial Manager. 
Potential Copyright Issues:
i) Figures 2B, 4B, 4C, 4D, 5, S1, and S2. Please (a) provide a direct link to the base layer of the map (i.e., the country or region border shape) and ensure this is also included in the figure legend; and (b) provide a link to the terms of use / license information for the base layer image or shapefile. We cannot publish proprietary or copyrighted maps (e.g. Google Maps, Mapquest) and the terms of use for your map base layer must be compatible with our CC BY 4.0 license.
Note: if you created the map in a software program like R or ArcGIS, please locate and indicate the source of the basemap shapefile onto which data has been plotted.
If your map was obtained from a copyrighted source please amend the figure so that the base map used is from an openly available source. Alternatively, please provide explicit written permission from the copyright holder granting you the right to publish the material under our CC BY 4.0 license.
If you are unsure whether you can use a map or not, please do reach out and we will be able to help you. The following websites are good examples of where you can source open access or public domain maps:
* U.S. Geological Survey (USGS) - All maps are in the public domain. ([http://www.usgs.gov](http://www.usgs.gov/))
* PlaniGlobe - All maps are published under a Creative Commons license so please cite u201cPlaniGlobe, [http://www.planiglobe.com](http://www.planiglobe.com/), CC BY 2.0u201d in the image credit after the caption. (<http://www.planiglobe.com/?lang=enl>)
* Natural Earth - All maps are public domain. (<http://www.naturalearthdata.com/about/terms-of-use/>).

**Response to Journal Requirements, F:** Regarding Fig. 2B, 4B, 4C, S1, these are solely shapefiles produced by the authors. We have referenced the online source that was used as a baseline for tracing the boundaries in the figure legends.

Regarding Fig. 4D and 5A, we have referenced the cartographer who created the historic map in the figure legends.

Regarding Fig. 5B and 5C, we have referenced the DTM data source, providing links in the caption for the data and the license. We have also referenced and provided the link for the ArcGIS Pro basemap.

Regarding Fig. S2, the reference has been provided in the figure caption.

Please see ‘Response to Journal Requirements, C’ regarding the copyright symbol that was previously in Figure 5 – this has now been removed.

All references are also included in the References list.

**Journal Requirements, G:** 7) In the online submission form, you indicated that The data in this study are available from the corresponding author upon reasonable request.. All PLOS journals now require all data underlying the findings described in their manuscript to be freely available to other researchers, either
1. In a public repository
2. Within the manuscript itself
3. Uploaded as supplementary information.
This policy applies to all data except where public deposition would breach compliance with the protocol approved by your research ethics board. If your data cannot be made publicly available for ethical or legal reasons (e.g., public availability would compromise patient privacy), please explain your reasons by return email and your exemption request will be escalated to the editor for approval. Your exemption request will be handled independently and will not hold up the peer review process, but will need to be resolved should your manuscript be accepted for publication. One of the Editorial team will then be in touch if there are any issues.

**Response to Journal Requirements, G:** The necessary data and shapefiles that underlie our findings are to be uploaded to GitHub.

**Journal Requirements, H:** 8) Please amend your detailed Financial Disclosure statement. This is published with the article. It must therefore be completed in full sentences and contain the exact wording you wish to be published.
1) State what role the funders took in the study. If the funders had no role in your study, please state: "The funders had no role in study design, data collection and analysis, decision to publish, or preparation of the manuscript."
2) If any authors received a salary from any of your funders, please state which authors and which funders..
If you did not receive any funding for this study, please simply state: u201cThe authors received no specific funding for this work.u201d

**Response to Journal Requirements, H:** The Financial Disclosure statement has been amended.

**Journal Requirements, I:** 9)  Please ensure that the funders and grant numbers match between the Financial Disclosure field and the Funding Information tab in your submission form. Note that the funders must be provided in the same order in both places as well.  

**Response to Journal Requirements, I:** The funders and grant numbers match between the Financial Disclosure field and the Funding Information tab.

**Reviewers' Comments**

Reviewer's Responses to Questions

**Key Review Criteria Required for Acceptance?**

As you describe the new analyses required for acceptance, please consider the following:


**Methods**

-Are the objectives of the study clearly articulated with a clear testable hypothesis stated?

-Is the study design appropriate to address the stated objectives?

-Is the population clearly described and appropriate for the hypothesis being tested?

-Is the sample size sufficient to ensure adequate power to address the hypothesis being tested?

-Were correct statistical analysis used to support conclusions?

-Are there concerns about ethical or regulatory requirements being met?

**Reviewer #1:** a) Objectives are well articulated with a clear testable hypothesis.
b) The study design was suitable elaborated to reach the stated objectives,
c) The population was described appropriately considering the historical records,
d) As far as authors were aware, the estimated sample was acceptable to address the hypothesis,
e) I am not familiar with all the statistical techniques the authors used, however they are well referenced.
f) ethical requirements were met.

**Response to Reviewer #1, Methods:** We thank the reviewer for the comments and we are glad that these requirements are met.

**Reviewer #2:** Yes,

**Response to Reviewer #2, Methods:** Thank you.

**Reviewer #3:** I believe the objectives were proposed in alignment with the availability of data and the possibilities for analyzing them, and the study was designed to address these objectives. The population is clearly described.

**Response to Reviewer #3, Methods:** Thank you.

**Reviewer #4:** The proposed objectives are clearly articulated at the end of the introduction.

Regarding the hypothesis to be tested, it appears that the authors posited that the apllication of modern spatial epidemiological methods to historical data would enable a deeper understantading of past and current wellow fever virus infection within the framework of human-environment interactions.

The population affected by yellow fever, as well as the cases extracted from the 1859 report of the Extraordinary Council of Public Health of Portugal,is clearly described in the Materials and Methods section. It is also clear where the environmental and socioeconomic data were obtained from.

The statiscical power of the sample is inherently limited by the historical data available for the analysis conducted. And yes, the statistical inference supports the conclusion.

All items in the Materials and Methods section are well described an clearly explained. The supplementary figures and tables ensure a clear understanding o the study area and the census data.

**Response to Reviewer #4 Methods:** We thank the reviewer for the comments. We can confirm that the hypothesis identified by the reviewer is correct.

**Reviewer #5:** The objectives of the study are clearly articulated. The methodology and analysis adequately described. No ethical or regulatory requirements was needed as the study is based on archived documentations.

**Response to Reviewer #5, Methods:** Thank you.

**Results**

-Does the analysis presented match the analysis plan?

-Are the results clearly and completely presented?

-Are the figures (Tables, Images) of sufficient quality for clarity?

**Reviewer #1:** Yes, the analysis presented match the analysis plan.
Results were clearly and well presented,
The figures are of sufficient quality and clearly shown.

**Response to Reviewer #1, Results:** Thank you.

**Reviewer #2:** Yes,

**Response to Reviewer #2, Results:** Thank you.

**Reviewer #3:** Results are clearly presented and match the methods, and they are sufficient to answer the objectives.

**Response to Reviewer #3, Results:** Thank you.

**Reviewer #4:** The results are consistent with the proposed objectives. The figures, which include graphs, diagrams, maps, as well as the information from the supplementary material. are clear and highly valuable for understanding the results present.

The authors appear to discuss the results lines 283-285, 288-290, 305-307, 323-324, 380-381, 421-423, 445-447, and 453-456. I suggest that these observations would be more appropriately presented in the Discussion section.

On line 299, the authors should specifiy the species Aedes aegypti rather than just the genus. The genus includes other species and does not accurately represent the vector species.

**Response to Reviewer #4 Methods:**

Thank you for these suggestions on moving materials into the Discussion. We have now moved each of the mentioned lines to the Discussion. Most notably, the reconciliation of lines 283-285, 288-290, and 323-324 have inspired a new paragraph in the Discussion that reads,

Page 19, lines 565-573: “Investigating the likely source of YF introduction to Lisbon in 1857 is of importance, providing insights into the mobility of the virus. While the RMS Tamar was the only ship to report YF cases in 1857, other ‘suspicious’ ships had been noted in the lazaretto (maritime quarantine first instated in Lisbon in 1837; see Esteves, 2014). Although it was not possible to assign the cause to one ship due to many ships not reporting illness to avoid sanitary measures, competent *Aedes aegypti* were likely repeatedly introduced during this process (Esteves, 2014). YF was able to become established in Lisbon; the infection attack rate was at least 10.3% of the population, however it was implied that cases could have totalled closer to 18,000 across the course of the epidemic, reaching 13.5%.”

Lines 305-307 have been moved to the Discussion, tying the discussion on R0/Rt together (“Franzini reported that 1857 had been a relatively ‘healthy’ year until August, with a lower mortality rate than previous years (Health Council, 1859).”). Lines 380-381 have been moved to the Discussion paragraph focusing on gender disparity in home vs hospital treatment. Lines 421-423 have been moved to the Discussion paragraph on the significance of proximity to the focal point of the epidemic, whilst making it clear that proximity is not the sole driver. Lines 445-447 have been moved to bolster the Discussion point on proximity wind buffers. Lines 453-456 have been moved to the Discussion point regarding the influence of elevation.

We have also amended Line 299 to specifically state “*Aedes aegypti”*, rather than only Aedes.

**Reviewer #5:** The results presented match the analysis plan.

**Response to Reviewer #5, Results:** Thank you.

**Conclusions**

-Are the conclusions supported by the data presented?

-Are the limitations of analysis clearly described?

-Do the authors discuss how these data can be helpful to advance our understanding of the topic under study?

-Is public health relevance addressed?

**Reviewer #1:** The conclusions are supported by records from the nineteen century in other cities.
Limitations of the study are well reported, the reliability of historical records were affected by the lack of data collection methods at the time and that should have influenced the epidemiological analysis.
The authors considered these data are helpful to improve the comprehension of yellow fever dynamics.Public health is considered through the records of Public Health Council. These records contributed much to describe the yellow fever outbreak in 1857 in Lisbon.

**Response to Reviewer #1, Conclusions:** Thank you.

**Reviewer #2:** Yes,

**Response to Reviewer #2, Conclusions:** Thank you.

**Reviewer #3:** Not entirely, but I suggest that the authors revise their conclusions to better align them with the reviewers' suggestions, should they decide to incorporate those suggestions.

**Response to Reviewer #3, Conclusions:** Thank you for the feedback. We have revised our conclusions appropriately to align with the changes made resulting from the reviewer comments. Based on the reviewers’ suggestions on the role of places of congregation on transmission and the value of such a study on present day prevention efforts, it now reads:

Page 26, lines 851-869: “By digitalising and mapping archival disease data, we demonstrated that the environment, combined with sociodemographic factors, contributed to the progression of the 1857 YF epidemic in Lisbon. Aligned with previous studies, our estimates of R0 illustrates that YFV is capable of rapid spread and decline predating vaccination. While the 1859 Health Council in Lisbon identified wind direction as the primary driver of YF, our findings highlight the complex interaction of human-environmental relations in shaping the epidemic. Out of the 34 parishes in urban Lisbon, our hot spot analysis illustrated a cluster of 15 high-risk parishes near the coastline. We found that the highest number of deaths occurred within connected streets confined in low-lying, built-up areas, illustrating the potential role of wind aiding mosquito distribution and concentration of mosquitoes near homes. Considerations of the role of places of congregation on YF transmission inform vector control programs, illustrating the transmission potential in public spaces, rather than predominately homes. Gender disparities existed in the reporting of YF fatalities. Although working-aged men accounted for most YF fatalities, the highest probability of death was among “non-designation” occupation individuals, mainly women working at home. Examining a historical urban epidemic in Europe provides a unique insight into how reemergence of YF in Europe may look amid climate change and growing interconnectivity. This study demonstrates the complexity of YFV transmission in a historic urban context and illustrates the need for continued research to understand YFV transmission and its spatial dispersal.”

**Reviewer #4: (A)** The conclusions are supported by the results, and the study limitations were clearly reported in lines 636 to 650 in the Discussion section.

The study presented is centainly relevant to Public Health,particularly regarding the transmission of urban yellow fever.

I did not find in the discussion a clear explanation of how the data can be usefull for advancing our understanding of the tropic under study. Except for its contribution to the growing body of literature on the reproduction number of the yellow fever virus including the identification of chances in the Rt variable over time during the course of the epidemic.

**Response to Reviewer #4, Conclusions - A:** Thank you for raising this point. We have added an additional paragraph towards the end of the Discussion that clarifies how the data and analysis can be used for advancing our understanding of the topic. We emphasise that our study provides a unique insight into a pre-vaccination epidemic, in a non-endemic region, in the wake of YF’s recent return to Europe.

We also highlight that the study has advanced our understanding of how sociodemographic and environmental factors drive YF, and how these factors interact with each other. The addition to the manuscript now reads,

Page 26, lines 818-833: “Our study offers a uniquely detailed insight into the impact of YF in the absence of a vaccine and illustrates how social (e.g. gender, living spaces, water supply and drainage, and occupations) and environmental factors (e.g. wind, elevation, temperature, and precipitation) drive outbreaks, working synoptically. The particular case of wind as a factor has been explored in terms of distance within an urban and historic setting, which adds to our understanding of the influence of wind on the transmission of YF, contributing to limited research on the topic. Further, examining an epidemic in a non-endemic region provides a strong example of the mobility of YF, and how vectors can migrate over distances much further than nature would allow, and still become established. This assessment is particularly imperative amid current discourses that YF has the potential to return to Europe as environmental conditions become more favourable and cross-continental travel increases in frequency and speed from endemic regions, increasing the chance that YF is imported to Europe (Javelle et al., 2019). Increasing our understanding of how YF has previously interacted with Europe means that we can better prepared and more resilient to YF’s return; the use of historical analyses can be applied to future projections, identifying areas likely to become suitable for *Aedes aegypti* establishment (Wint et al., 2022).”

**(B)** Based on the results obtained and comparisons with other studies, I ask ifthe the authors could discuss how wind and altitude may currently influence the dispersal or the vector and the urban yellow fever virus within the same neighborhoods in lisbon Portugal.In other word, such an internal extrapolation could be considered in light of present urbanization patterns and environmental conditions.

**Response to Reviewer #4, Conclusions - B:** Thank you for this suggestion. We have added a new paragraph in the Discussion section which considers the impact of wind, elevation, and urbanisation in the context of present-day Lisbon. On page 21, lines 659-666, we discuss how elevation drives differences in urbanisation, which in turn changes the landscape. According to da Silva et al., (2025), a difference in landscape results in more/less connectivity for humans, either connecting/disconnecting them from the rest of the population, which would impact YF. The change in landscape impacts wind; a less structured urbanisation creates a more fragmented urban landscape, which reduces the generation of wind tunnels in roads between buildings, which can play a role in the dispersal of vectors. We also touch on the modern urbanisation of the parish of Santa Maria Maior, which replaced the worst YF-affected parishes, and how this may perpetuate urban transmission cycles that have been evidenced to cause high rates of infection, particularly in optimal geographic locations. The addition to the manuscript reads,

Page 21, lines 658-673: “Our findings on the impact of elevation and wind in relation to the physical environment allows insight into how the environment may shape YF in present-day Lisbon. Lisbon’s urban development is greatly dictated by its natural boundaries and geomorphological conditions (Morgado, 2023). The present-day parish of Marvila sits on the coast with significant elevation change, which results in a less compact urban morphology, with buildings irregularly covering the landscape (da Silva et al., 2025). In the context of YF’s potential return, this parish may experience greater YF burden where elevation is lower and houses are more concentrated, while the more fragmented urban landscape means that there are weaker transport connections, slowing the movement of people (da Silva et al., 2025), and vectors via wind tunnels. Further, the present-day parish of Santa Maria Maior, an amalgamation of the former historic centre that included the worst YF-affected parishes in 1857 (such as Sé, Magdalena, and São Miguel) has undergone mass touristification (Martins, 2022). Along with the commodification of places of historic culture, this parish has been urbanised, particularly with short-term rentals (Rodrigues et al., 2022; Martins, 2022). Given the rate of urbanisation in locations evidenced to have optimal environmental conditions for YF, it can be inferred that similar urban transmission cycles could occur in the future, putting the same areas at risk.”

**Reviewer #5:** Overall the conclusions are supported by the presented data. However as mentioned in the section below more emphasis and clarification are needed on certain aspects

**Response to Reviewer #5, Conclusions:**

Thank you – we have addressed this directly, specifically in ‘Response to Reviewer #5, Editorial and Data Presentation Modifications’ A- F.

**Editorial and Data Presentation Modifications**

Use this section for editorial suggestions as well as relatively minor modifications of existing data that would enhance clarity. If the only modifications needed are minor and/or editorial, you may wish to recommend “Minor Revision” or “Accept”.

**Reviewer #1:** No suggestions.

**Response to Reviewer #1, Editorial and Data Presentation Modifications:** Thank you.

**Reviewer #2:** (No Response)

**Response to Reviewer #2, Editorial and Data Presentation Modifications:** Thank you.

**Reviewer #3:** I will highlight here the suggestions and questions that are more specific to certain sections of the text, and in the following box some more general comments and suggestions. I would like a response from the authors regarding each of my comments:

**(A)** 1) Line 46 – Please clarify the statement: “...Aedes (Stegomyia) aegypti, which can circulate in both rural and urban areas (Gabiane et al., 2022).” I believe this is a generalization that leaves room for doubt regarding what exactly is meant by “circulation of A. aegypti in rural areas.” For example, this does not seem applicable to the situation of yellow fever circulation in the Americas. Do the authors have a different view? Could they comment/clarify?

**Response to Reviewer #3, Editorial and Data Presentation Modifications - A:** Thank you for raising this point. We have amended the statement to,

Page 2, line 63-66: “…*Aedes (Stegomyia) aegypti*, which predominantly circulate in urban areas, but can also, and are increasingly, invading rural areas (Fikrig et al., 2025), perpetuating yellow fever (YF) via the intermediate and urban transmission cycles (Gabiane et al., 2022).”

We have included a new reference to Fikrig et al., (2025), who examine the rise of *Aedes aegypti* in the rural Peruvian Amazon. Although YF in the Americas is predominantly driven by sylvatic mosquitoes, such as *Haemogogus* and *Sabethes*, *Aedes aegypti* are becoming increasingly prevalent in these rural areas. We do also acknowledge that *Aedes aegypti* are predominantly urban vectors, which is important to highlight given the context of the Lisbon epidemic.

**(B)** 2) Line 48 – Have the authors considered adopting the new designation “Orthoflavivirus,” according to Postler TS, Beer M, Blitvich BJ, et al. Renaming of the genus Flavivirus to Orthoflavivirus and extension of binomial species names within the family Flaviviridae. Arch Virol. 2023;168(9). doi:10.1007/s00705-023-05835-1, or is some other option being evaluated?

**Response to Reviewer #3, Editorial and Data Presentation Modifications - B:** Thank you for highlighting the new designation of Orthoflavivirus. We have now incorporated this term on line 78 and cited the given article by Postler et al, (2023).

**(C)** 3) Line 59 – If applicable, I suggest using the original reference: Bryant JE, Holmes EC, Barrett ADT. Out of Africa: A molecular perspective on the introduction of yellow fever virus into the Americas. PLoS Pathog. 2007;3(5):0668-0673. doi:10.1371/journal.ppat.0030075

**Response to Reviewer #3, Editorial and Data Presentation Modifications – C:** Thank you for highlighting this paper. We have now altered line 79 to cite Bryant et al (2007).

Page 3, line 79: “YFV may have originated between 1,500 and 3,000 years ago in Central Africa (Bryant et al., 2007; Monath and Vasconcelos, 2015), before diverging into four genotypes […]”.

**(D)** 4) Line 61 – “YFV epidemic,” when the correct term should be “YF epidemic.” Please check the rest of the text, as this occurs repeatedly.

**Response to Reviewer #3, Editorial and Data Presentation Modifications – D:** Thank you for highlighting this. We have now reviewed the manuscript and altered references to YFV and YF appropriately.

**(E)** 5) Lines 61–62 – Please check whether the following references might be earlier and therefore more appropriate:
• Bryant JE, Holmes EC, Barrett ADT. Out of Africa… (2007)
• Klitting R, Gould EA, Paupy C, de Lamballerie X. What does the future hold for yellow fever virus? (I). Genes (Basel). 2018;9(6). doi:10.3390/genes9060291

**Response to Reviewer #3, Editorial and Data Presentation Modifications – E:** Thank you for highlighting these references. We have added the citation of Klitting et al., (2018) on Line 84. Bryant et al., (2007) has been referenced on Lines 79 and 86.

**(F)** 6) Line 74 – Do the authors consider the 1856 Lisbon epidemic to be a separate epidemic? What comments could they offer about the immune individuals generated by that epidemic and their relevance (or lack thereof) during the 1857 epidemic?

**Response to Reviewer #3, Editorial and Data Presentation Modifications – F:** Thank you for raising this point. Due to the relatively small scale of the epidemic in 1856, we do not believe that immunity generated by infection from that epidemic would have had much significance/relevance to the outcomes of the 1857 epidemic. Given that there was an increase from 87 fatalities in 1856 to 5,652 fatalities in the same city, but only the following year, it does not seem that 1856-derived immunity offered any protection of significance, given the scale of the later epidemic.

We have added an additional statement to Page 3, lines 95-97 of the manuscript to clarify these thoughts, which reads “Given that the 1856 Lisbon epidemic had little impact on the city, compared to that in 1857, it is highly unlikely that any immunity occurring from infection would have had a significant impact on the outcomes of 1857.”

**(G)** 7) Lines 78–80 – I suggest strengthening the justification for choosing this particular outbreak. If the cited reference provides more data on magnitude, for example, it would be important to mention this.

**Response to Reviewer #3, Editorial and Data Presentation Modifications – G:** We have added additional justifications in the Introduction to clarify why this particular epidemic was chosen, indicating the scale of the epidemic and the breadth and detail of the data reported by the Health Council. Analysis of this epidemic is particularly important in the face of YFV’s potential to continue migrating northwards, back into Europe.

Page 3, lines 100-103, “Although a recurring burden to Lisbon during the eighteenth and nineteenth centuries, the scale of the 1857 YF epidemic, and the subsequent breadth of data reported on such magnitude by the Health Council warrants an in-depth analysis of the epidemic, particularly in light of the potential return of YF and arboviruses to Europe.”

Page 5, lines 149-157, “Our analysis using nearly complete data of a relatively unstudied urban epidemic of great magnitude in 1857, in a region that is non-endemic to *Aedes aegypti*, and consequently YF, provides a strong opportunity to assess the drivers of YF. The 1857 Lisbon epidemic is a seminal example illustrating the relationship that YF has previously had with Europe, and may also have in the future as climate change intensifies. Therefore, it is important to understand how YF may spread in a European setting to support the development of epidemic preparedness strategies. These findings using modern spatial epidemiological methods can help inform our understanding of past and present YF infections amid present-day climate and public health challenges.”

**(H)** 8) Lines 82–83 – What is the rationale behind this statement? Did merchant ships departing from Brazil facilitate new introductions of the virus into Lisbon? This is not clear.

**Response to Reviewer #3, Editorial and Data Presentation Modifications – H:** We have clarified the rationale behind the link between Portugal-Brazil travel and trade and the mobility of vectors. Given the growing interconnectedness between Brazil and Portugal, with the vectors endemic to the former, this enabled the transportation of vectors over longer distances, infecting non-endemic regions.

We have added to the manuscript to clarify this rationale.

Page 4, lines 118-127: “Portugal-Brazil migration and trade intensified in the 1840s (Chalhoub, 1993), contributing to the ignition of YF outbreaks in Lisbon by mobilising vectors from endemic regions of South America (Esteves, 2014). The importation of YF from Brazil to Portugal was formally recognised by the King of Portugal for causing the 1856 Porto outbreak; Brazil also drove YF to Montevidéo, Uruguay, and Buenos Aires, Argentina in 1857 (Franco, 1969), illustrating the reach of YF from Brazil. The advent of steam power in the 1830s halved transatlantic travel time to 15 days (Harrison, 2013), which fell within the three-to-five-week lifespan of *Aedes aegypti*, meaning that the vectors could survive the transatlantic journey by breeding within water-containing objects onboard (Maricopa County Environmental Services, 2006).”

**(I)** 9) Lines 242–243 – The “analysis” at the time suggested a relationship between wind (or wind direction) and the spread of cases. Keep in mind that this “conclusion” was likely based on the idea of miasmas and not on vector-borne transmission. Where is this historical “analysis” linking wind described? Considering that Aedes is a mosquito that lives indoors, how do the authors envision such strong influence of wind?
In some situations, wind has been considered important for the dispersal of sylvatic vectors involved in yellow fever transmission in the Americas. I do not believe this hypothesis can be directly applied to urban transmission by Aedes, particularly when analyzing data from an epidemic that occurred 168 years ago.

**Response to Reviewer #3, Editorial and Data Presentation Modifications - I:** We acknowledge that at the time of the epidemic, the researchers would not have known about vector-borne transmission given that it was not proven for YF until 1881 (Rodhain, 2022). The authors of the report state “If yellow fever is important, […], it is because it is transmitted by individuals and objects, and therefore must be considered contagious.” This would suggest that the authors were not biased towards miasma theory, which was relating to ‘bad air’.

The historical “analysis” refers to what’s being quoted from the historic report, “Of all the meteorological elements, the one with the greatest relationship with the epidemic force is the direction of the winds. If one examines the line of the winds, it is seen that the decline in the number of those attacked often coincides with the winds from the north, north-northwest, to northeast quadrant.” While the basis of this claim may simply be coincidental, it is an interesting point given that decline in cases coincides with winds blowing towards the coast, and thus, not perpetuating the dispersal of them from the source/highest density.

We view wind as having an influence as a passive transport mechanism, aligning with Marcantonio et al. (2019) who concluded that wind contributed to the involuntary movement of *Aedes aegypti* in the urban area of Madera, California. We acknowledge that *Aedes aegypti* typically reside indoors, however, they must get to these locations first from their source at customs after having been imported. Although there are perhaps other modes of distributing mosquitoes, such as transporting water barrels with vectors harbouring inside, there is evidence that wind is another plausible driver, supported by researchers such as De Almeida (2019) and Yamashita et al. (2018).

Further, there are cases of *Aedes aegypti* being partially exophilic too (Facchinelli et al., 2023; Konkon et al., 2025). Though the vectors focus around homes, they may also reside outside, near homes. These conditions mean that they would be vulnerable to the influence of wind.

Ultimately, *Aedes aegypti* mostly circulate and bite indoors, but there are opportunities for them to be exposed to wind. Further, according to the historic report, ventilation of housing was recommended (such as windows and doors open) during the epidemic and was considered the best means of ‘disinfection’. While the original statement may have been influenced by the miasma theory, the ventilation of buildings may have aided in expelling some vectors from buildings, through natural draughts during windy conditions, further exposing them to the outdoors.

**(J)** 10) Lines 440–447 – It does not seem that the data collected provide enough support to conclude that viral dispersal occurred primarily due to mosquito movement rather than the movement of infected people. Furthermore, the role of places of congregation—such as churches, public areas, taverns, and other meeting points—was not adequately addressed. Although these were included in the analyses, it seems the authors treated these locations as dispersion points from which infected mosquitoes were transported to other areas, rather than as venues visited by people who could become infected there.

**Response to Reviewer #3, Editorial and Data Presentation Modifications - J:** Thank you for raising this point. We have added a new paragraph into the Discussion that considers the role of places of congregation in YF transmission. The popularity of taverns among men in 19^th^ century Lisbon may have contributed to the higher proportions of infections and fatalities in men, the mobility of individuals to these social, public places, may have put them at-risk of infection from vectors residing in these buildings.

The addition to the manuscript now reads,

Page 22, lines 675-703: “Along with the mobility of vectors it is important to consider the mobility of individuals in contributing to the widespread transmission of YF. Places of congregation, such as taverns, churches, and schools, would have been suitable sites for *Aedes aegypti* to reside in or nearby, as they provide abundant opportunities for blood meals; this was demonstrated with the case of schools (García-Rejón et al., 2011) and catholic churches in Merida City, Mexico (Baak-Baak et al., 2018). In these locations, transmission occurs independent of vector mobility. Historically, and more comparatively to Lisbon in 1857, Sims (2020) reports that 40-50% of 2,000 fatalities during the 1873 Memphis, USA, YF epidemic attended the same church (St. Brigid’s Catholic Church). Though another driver may have been involved in causing so many fatalities in parishioners from this church, such as individuals living locally and close together, it is likely that the church acted as a hotspot for infections. The vectors were present in a public place where individuals congregate, providing a plentiful blood source. In nineteenth century Lisbon, taverns were a place where large groups would congregate, of which a large proportion were men; there were at least 1,556 ‘drinking establishments’ in 1825, which illustrates the prevalence of this form of public space in Lisbon (Lousada, 1997). Further, boarding houses that primarily provided temporary or semi-permanent accommodation for men were often located towards the coast in the parish of Rocio (Health Council, 1859), where the accommodation would be close to places of work, such as customs and industry. The aggregation of individuals in these boarding houses in the worst-affected neighbourhood, in an optimal geographic location for YF, may have acted as an opportunity for infection from vectors, as was observed during eighteenth and nineteenth century YF epidemics in New York, USA (Lasdow, 2022). Given that men accounted for most cases and fatalities during the 1857 YF epidemic, it is likely that many of these infections occurred in androcentric places of congregation. Our research contributes to limited literature on the role of places of congregation in YF transmission. While contemporary studies mainly focus on YF transmission in rural or forested areas (Prado et al., 2024; Kersul et al., 2024), our findings unveil how YF can spread in dense public spaces and can inform vector control programs, focusing on public places, rather than the home. Future research efforts could map and analyse the spatial relationships between YF burden and places of congregation.”

**(K)** 11) Lines 499–500 – As highlighted by the authors themselves, I believe this point is debatable and deserves further discussion.

**Response to Reviewer #3, Editorial and Data Presentation Modifications - K:** We have expanded on this point, clarifying that winds blowing from the north (from inland, towards the coast), may hinder the dispersal of vectors from their source at customs. At these times, there was not a backwind to propel vectors. Please see ‘Response to Reviewer #3, Editorial and Data Presentation Modifications – I’ for further details.

**(L)** 12) Lines 502–511 – Please carefully review the reference “Almeida, M. A. B., dos Santos, E., Cardoso, J. d. C., da Silva, L. G., Rabelo, R. M., and Bicca-Marques, J. C. (2019). Predicting Yellow Fever Through Species Distribution Modeling of Virus, Vector, and Monkeys. EcoHealth. 16: 95–108.” for two main reasons:
1. The cited study was conducted with sylvatic vectors in southern South America, in natural (non-urban) environments, and using contemporary data from the time of the event. The comparison does not seem directly applicable.
2. Note that the full text of the cited article states:
“However, we cannot reject the possibility that roads play a role in the dispersal of infected vectors either via transport in vehicles or the influence of wind ‘tunnels.’”
Yet the manuscript cites it as: “De Almeida (2019) highlighted the role that streets play in vector dispersal as they generate wind ‘tunnels’, funnelling mosquitoes into urban spaces. While wind facilitated vector dispersal, their establishment was likely also driven by high housing density and the abundance of stagnant water in these areas.”

**Response to Reviewer #3, Editorial and Data Presentation Modifications – L:** Thank you for your comment. While we acknowledge that contemporary conditions are different between the focus of the referenced paper and our manuscript, we wanted to illustrate the role that roads/streets may have in facilitating dispersal. There is limited research into this area, particularly into historical and European contexts, hence the use of this reference. De Almeida et al., (2019) stated that roads may play a role in the dispersal of infected vectors by generating ‘wind tunnels’. The streets between buildings essentially funnel wind in a given direction, in ‘tunnels’ through the streets. Therefore, vectors that are outside may be dispersed through these routes, funnelling vectors by buildings on streets where they may perpetuate infections. We have added support for this statement with reference to Yamashita et al. (2018) and Marcantonio et al. (2019) who demonstrate the assistance of wind in dispersing *Aedes aegypti* in an urban setting. The addition to the manuscript reads on page 20, lines 618-621: “This is supported by Yamashita et al., (2018) who demonstrated that wind assisted the urban distribution of *Aedes aegypti* in two Brazilian cities, and Marcantonio et al. (2019), who concluded that wind contributed to involuntary movements of *Aedes aegypti* in the city of Madera, California.”

**(M)** 13) Lines 513–533 – There may be differences in vector density and perhaps less human movement due to the topography (hills). In other words, higher-altitude areas might have fewer mosquitoes and fewer people moving around; individuals may have been infected in lower areas (where rainwater accumulates), and then, returned to higher areas where they could infect mosquitoes already present there, after teh incubation period.

**Response to Reviewer #3, Editorial and Data Presentation Modifications – M:** Thank you for raising this point. We have restructured, and added to, the Discussion paragraph on the impact of elevation. We highlight the change in human and vector densities at varying elevations, also highlighting that the accumulation of rainwater at low altitudes is a factor in driving higher vector densities. We have also acknowledged the relationship that humans and vectors have in changing elevation; i.e. people can travel to lower altitudes, become infected, and return to high altitudes to continue the transmission cycle.

It now reads on page 20, lines 630-656: “Elevation was also found to be related to the distribution of YF, not only indirectly through influencing the distance at which wind can disperse vectors but also acting as a natural barrier. Vectors do not travel far from their hatch sites without the influence of wind but can be barred from ‘climbing’ over higher elevations (Newman et al., 2024). Our results suggest that the hills to the west of the city seemingly shielded the neighbourhood of Alcantara, leaving it far less affected than during past outbreaks of other diseases, such as cholera (Health Council, 1859). The hills to the west, dividing the centremost part of the city from Alcantara, provided a barrier to infection where conditions may otherwise have been optimal for transmission; Alcantara neighbourhood includes the three most populous parishes. Generally, we found that people living at higher elevations were at a lower risk of death from YF; few deaths occurred on streets with elevation above 80 m overall. This, however, could have been due to lower population density at higher elevations. Though Santa Catharina, of higher elevation at 40 m altitude, still suffered more than São Paulo. Higher altitude areas would likely have had less movement of both people and vectors; individuals may have been infected in lower areas, and returned to higher areas to continue the transmission cycle. Additionally, the parish with the highest average altitude, Santa Cruz do Castello, only suffered 19 fatalities, but given its small population the fatality rate still reached 3.3%. Located near the coast and thus in an optimal location for YF exposure, Santa Cruz do Castello is a hotspot due to its surrounding parishes but was the 31st parish to be infected. This indicates a clear delay in the parish being impacted by the epidemic, despite being near the worst affected parishes that were infected during the early onset stages of the epidemic. The densely populated streets with less than 20 m in altitude were within proximity to customs, primarily housing customs employees, and were the worst affected streets. This aligns with the findings of Meena (2020), who stated that lower-lying urban areas perpetuate the spread of *Aedes* mosquitoes, in part due to the accumulation of rainwater at low altitudes, and therefore vector breeding sites.. This illustrates that wind and elevation may work synoptically in driving YF by determining the spatial variation in vector distribution.”

**(N)** 14) Lines 535–537 – I consulted the article by Newman et al. (2024) (although I did not read it thoroughly, so I may have missed something), and I did not find this statement directly: “staying within 30–60 metres of their hatch site in the absence of wind.” Could the authors please clarify?

**Response to Reviewer #3, Editorial and Data Presentation Modifications – N:** I quote from Newman et al., (2024): “Adults generally do not disperse beyond 30–60 m from their hatch site and tend to cluster around homes, but will rarely disperse as far as 500 m for oviposition sites.” This illustrates that *Aedes aegypti* do not actively travel far from their hatch sites around homes (within 30-60m), and so without the action of wind, this is where they will stay. Wind, however, adds a dynamic whereby mosquitoes may be transported passively.

**(O)** 15) Lines 660–662 – I would like to know more about this conclusion:
“We found that the highest number of deaths occurred within connected streets confined in low-lying, built-up areas, illustrating the potential role of wind aiding mosquito distribution and concentration of mosquitoes near homes.” Once again, Aedes aegypti is a mosquito that lives indoors. It does not need wind to remain in these environments. When food sources (people) and oviposition sites (small water collections) are available, mosquitoes remain in place. Furthermore, I insist: what evidence do we have about the influence of wind inside homes in terms of carrying mosquitoes over certain distances?

**Response to Reviewer #3, Editorial and Data Presentation Modifications – O:** Please refer to the ‘Response to Reviewer #3, Editorial and Data Presentation Modifications - I:’, where we acknowledge that while *Aedes aegypti* typically reside indoors, there are potential opportunities for them to become exposed to wind.

We have expanded on our Discussion on wind as a driver with the following addition on page 20, lines 610-621, “Although *Aedes aegypti* are typically endophilic (rest and bite indoors), there are opportunities for the vectors to be involuntarily moved by wind as a passive transport mechanism when settled near homes, but outside; there are cases of *Aedes aegypti* being partially exophilic (Facchinelli et al., 2023; Konkon et al., 2025). Further, the vectors must be dispersed from the source at customs after importation before they are able to circulate near buildings. While this could be from the transportation of water barrels or luggage with vectors harbouring inside, wind may also play a role in this dynamic. De Almeida (2019) highlighted the role that streets may play in vector dispersal as they generate wind ‘tunnels’, funnelling mosquitoes into urban spaces. This is supported by Yamashita et al., (2018) who demonstrated that wind assisted the urban distribution of *Aedes aegypti* in two Brazilian cities, and Marcantonio et al. (2019), who concluded that wind contributed to involuntary movements of *Aedes aegypti* in the city of Madera, California”.

**Reviewer #4:** I recommend a less thorough review for approval, in accordance with the observations made in the aforementioned items.

**Response to Reviewer #4, Editorial and Data Presentation Modifications:** Thank you – we have taken the observations into account and actioned where appropriate.

**Reviewer #5:** **(A)** Here is an excellent study using archive data to recreate a historical or deathly epidemic. The depth and breath of the analysis and methodology is worth appraising and congratulating. However, there are, minor issues that need to be clarified prior to publication.
Overall, there is a lot of emphasis on the factors contributing to spread it will also be interesting to highlight if there are any factors that led to a decline of the epidemic.

**Response to Reviewer #5, Editorial and Data Presentation Modifications – A:** Thank you for the comments. We have now expanded the Results and Discussion sections to address possible factors that led to a decline in the epidemic.

In the Results section, on page 10, lines 345-351: “November 1857 experienced a particularly wet month, receiving 231 mm of precipitation, compared to only 4.2 mm of precipitation in November 1856. Further, temperatures began to decrease from the high averages observed in summer and autumn, dropping to an average temperature of 7.5°C in January 1858, and 11.3°C in February 1858. These conditions coincided with a decline in cases in all areas simultaneously from November onwards, until cases had disappeared entirely by February 1858.”

This addition to the Results section links with the new paragraphs added to the Discussion, which focus on the environmental factors that led to the decline of the epidemic.

The additions read on page 18, lines 541-563: “Cases of infection began to decline after reaching a peak on 20th October, coinciding with the onset of colder and wetter months. While storing rainwater encourages vector breeding to an extent by creating stagnant water pools (Haroon et al., 2020), there is less need for the long-term water storage which was used through the dry, summer months, and intense rainfall can wash away developing larvae from breeding sites (Newman et al., 2024). November 1857, which saw a large decline in cases, was particularly wet. A decrease in average temperatures from their peak in July 1857 (24.0°C) will have also contributed to a decline in *Aedes aegypti* populations as temperatures moved further away from the optimal temperature for offspring production (29.2°C) (Yang et al., 2009). Only temperatures above 12°C are suitable for population growth (Simoy et al., 2015) and monthly average temperatures dropped considerably below this in December 1857 (9.8°C). The number of cases continued to decline in all locations throughout the winter months, before ceasing to occur in February 1858. A simultaneous decrease in infection on the city scale suggests that the change in environmental conditions was a strong driver of decline, removing the optimal conditions for *Aedes aegypti* breeding.

It is worth noting that there was a possible population-based reason for the epidemic’s decline; by the end of November, the number of cases in the two worst affected parishes, Sé and Magdalena, had dropped drastically due to the high rate of population decline (Health Council, 1859). These parishes experienced the highest mortality rate so death and emigration had reduced the population vulnerable to new infections greatly (Health Council, 1859). Being in the optimal locations for YF, the new lack of infectivity potential will have caused a marked decline in overall infections.”

We believe that this suitably addresses both environmental and social determinants of epidemic decline.

**(B)** Secondly, contrary to available evidence, the authors highlighted in Line 478-492 that population density was not the primary driver of disease. Furthermore, in line 478-486. The least populated Rocio neighbourhood recorded the highest mortality rate. Was the Rocio neighbourhood least populous in terms of persons leaving in the area? Could it have been populous in terms of number of people working in the area?

**Response to Reviewer #5, Editorial and Data Presentation Modifications – B:** Thank you for raising this point. We have now added into the manuscript an acknowledgement of Rocio as a place for many people to work, even if they do not necessarily live there. We have made it clear that the geographical distribution of the population is an important driver of disease.

The new additions read on page 19, lines 583-591: “While Rocio was the least populous neighbourhood in number of people living there, Rocio was more populous in terms of the number of people working there. Rocio is in low-lying, central Lisbon nearby customs and industries where many worked. For example, there was a high fatality rate of the commercial occupation group, such as traders, who frequented the customs offices (Health Council, 1859). Zahouli et al., (2017) and Liu-Helmersson et al., (2019) highlight that a higher population (density) would increase exposure to infected mosquitoes and transmission. Whilst true, this cannot fully explain epidemic extent in Lisbon 1857. The geographical distribution of the population is demonstrated to be an important driver of disease.”

**(C)**Thirdly, line 159-160. Data on temp and precipitation were also retrieved, briefly mentioned in the results section but not discussed in great lengths as observed with the wind data. Considering the contribution of temperature is one of the study objectives, I will suggest for the inclusion of FigS3 as a main figure rather than a supplementary figure.

**Response to Reviewer #5, Editorial and Data Presentation Modifications – C:** We have expanded on the Discussion on temperature and precipitation with a new paragraph that discusses their relationship with a decline in the epidemic. Please refer to ‘Response to Reviewer #5, Editorial and Data Presentation Modifications – A’**.**

Unfortunately, due to limits on the number of figures and the current flow and organisation of the manuscript, we feel that Fig S3 fits best in the Supplementary Information. Further, temperature and precipitation are two drivers that commonly feature in literature, so we intend to draw attention to different foci, whilst still addressing temperature and precipitation as variables.

**(D)** Minor
1) Abstract: Line 18 rephrase Despite the availability of a highly effective vaccine, …

**Response to Reviewer #5, Editorial and Data Presentation Modifications – D:** Thank you for the suggestion. We have now rephrased the opening line of the Abstract.

**(E)** 2) Fig 1a – rephrase the statement “ The only ship to declare that YFV was on board.” I assume these are YF patients or suspected patients.
Line 282…declaring the presence of YF cases or patients onboard
Line 283 verify YFV in 1857 = report YF cases

**Response to Reviewer #5, Editorial and Data Presentation Modifications – E:** Thank you for the suggestion. We have now rephrased the above to specify YF patients/cases.

**(F)** 3) Line 328 Figure 1 rephrase legend” Timeline and characteristic of the 1857 Yellow fever epidemic. It will be great to also consider the reconsider the legend of the other figures precising it’s the 1857 Yellow Fever epidemic

**Response to Reviewer #5, Editorial and Data Presentation Modifications – F:** Thank you for the suggestion. We have now rephrased each figure caption, specifying that the figures relate to the 1857 YF epidemic in Lisbon.

**Summary and General Comments**

Use this section to provide overall comments, discuss strengths/weaknesses of the study, novelty, significance, general execution and scholarship. You may also include additional comments for the author, including concerns about dual publication, research ethics, or publication ethics. If requesting major revision, please articulate the new experiments that are needed.

**Reviewer #1:** The manuscript's title summarize the content and uses keywords to emphasize the occurrence of yellow fever outbreak in the portuary city, Lisbon, in urban space. As commented before to authors, lack of data collection methods should influence the epidemiological analysis. However, the discussion shows very support material and evidence arguments to demonstrate the dynamics of yellow fever at that that period at the city of Lisbon. There are no concerns about dual publication, research or publication ethics.

**Response to Reviewer #1, Summary and General Comments:** Thank you for the comments.

**Reviewer #2:** Re: Spatial and Social Determinants of the 1857 Yellow Fever Epidemic in Lisbon
Bates et al. conducted a digitisation of archival data and evaluated the influence of environmental and social factors on the yellow fever outbreak of that period. The study presents an engaging and insightful narrative that effectively demonstrates how digitising historical records can enhance our understanding of past public health events and yield valuable lessons for present and future disease control efforts. I read the article with great interest and found it both informative and enjoyable.
Discussion: This section is heavily dominated by the presentation of detailed historical data rather than by synthesising the key insights. It would benefit from a broader interpretation that highlights the main lessons learned and outlines potential strategies or scenarios to prevent the large-scale mortality and morbidity observed at that time.

**Response to Reviewer #2, Summary and General Comments:** We agree with the reviewer that the paper can benefit from a broader interpretation of the findings, and the main takeaways on both the past and present yellow fever outbreaks. In particular, we want to highlight how the findings from our study can help inform the literature on yellow fever (YF) outbreaks, both in the past and in present day.

In the first paragraph of the discussion, we have now added a summary of the key takeaways from the study, including how our findings can be applied to studying YF in the present day.

page 17, lines 516-526: “We combined historical YF case records with socio-environmental data to examine a major European outbreak in the pre-vaccination era, offering insights into how YF could re-emerge and spread in underexposed and unvaccinated populations. Our historical analysis provides a framework for interpreting YF transmission under comparable socio-environmental conditions today. This is particularly relevant amid concerns that YF could return to Europe as environmental conditions become more favourable and cross-continental travel from endemic regions increases in frequency and speed, raising the likelihood of importation (Javelle et al., 2019). By strengthening our understanding of how YF has previously interacted with Europe, we can improve preparedness and resilience to its potential re-emergence, and use historical evidence to inform future projections for *Aedes* suitability (Wint et al., 2022).”

Second, as part of our ‘Response to Reviewer #3, Editorial and Data Presentation Modifications – J’, we have expanded on the role of human mobility in places of congregation. In this paragraph, we specifically highlight the following:

Page 22, lines 675-703: Along with the mobility of vectors it is important to consider the mobility of individuals in contributing to the widespread transmission of YF… Our research contributes to limited literature on the role of places of congregation in YF transmission. While contemporary studies mainly focus on YF transmission in rural or forested areas (Prado et al., 2024; Kersul et al., 2024), our findings unveil how YF can spread in dense public spaces and can inform vector control programs, focusing on public places, rather than the home. Future research efforts could map and analyse the spatial relationships between YF burden and places of congregation.”

Next, in our paragraph on the gendered risks of YF, we have added the following sentences to show how recognising specific risk groups could have prevented mortality at the time, and that this should remain a priority in future research.

page 25, lines 787-797: “These factors, combined with traditional gender roles and limited healthcare access, shaped the risk of YFV-related deaths among women in 1857 Lisbon. Historically, recognising women as a high-risk demographic group could have enabled more targeted, efficient interventions, potentially preventing many avoidable deaths. Since spaces in society - from workplaces to homes - have been and continue to be, highly gendered, this raises the idea that YF itself is a “gendered disease” (Sommerseth and Walhout, 2022). Traditional gender roles affected the activities performed by men and women, influencing relative vulnerabilities to the disease and access to treatment. These features of gendered disease are still visible today in modern cases of arboviruses such as dengue (Akter et al., 2024), Zika (Coutinho et al., 2021), and chikungunya (Vidal et al., 2020), highlighting the pressing need to address the gendered dimensions of arboviral diseases.”

In our paragraph on vaccination, we now state that the apparent effectiveness of a vaccine at the time would have been a positive lesson for health professionals as they were able to gather evidence that vaccines could be of use against YF. This was particularly important given that the general consensus on the effectiveness of vaccines on severe illnesses was being questioned at the time. Further, we have also expanded this paragraph to reflect how unequal access to vaccination and broader healthcare inequities can shape health outcomes. We have now revised this paragraph and added the following sentences:

page 25, lines 799-816: “The Health Council reported the presence of a vaccine that, although deduced from a small sample size, seemed to prove somewhat effective in reducing fatalities. The Health Council is unclear as to the exact nature of the vaccine but, as no YFV vaccination had been produced until 1937 (Rodhain, 2022), the ‘vaccinated’ were likely protected against smallpox. Since vaccines were not freely available in Portugal until the National Vaccination Program began in 1965 (Fonseca et al., 2021), it is likely that vaccination in nineteenth-century Portugal required payment. Additionally, evidence suggests that more men than women were vaccinated, indicating that vaccine access may have already been a sociodemographic determinant of YF outcomes as early as 1857. Although it appears that the vaccine reduces mortality, our study suggests that it simply masks the real cause of reduced mortality, which is wealth and access. Those with the money to do so could remove themselves from the urban boundary, avoiding the reach of *Aedes aegypti*. In this sense, the “effect” of the vaccine may partly reflect social selection rather than protection alone. Nonetheless, in the broader context of nineteenth-century public health, these early reports were influential in shaping professional confidence that vaccination could be useful against other diseases at a moment when vaccine efficacy was actively debated (Health Council, 1859). This finding also points to an ongoing challenge in public health. During arbovirus outbreaks, intervention effectiveness can be overstated if analyses do not adequately address inequities.”

**Reviewer #3:** **(A)** I would like to commend the initiative taken by Dr. Li and her colleagues, which, in my view, represents an act of courage given the magnitude of the challenge. I consider this study to be a highly relevant effort by the authors. Revisiting historical yellow fever outbreaks and analyzing them in light of current knowledge is a very pertinent idea, one that may be necessary under certain circumstances and represents a creative way of reconstructing the history of the disease. This approach certainly required substantial research effort and meaningful collaborative work among the teams involved, especially considering the scarcity of data and information that characterizes certain historical periods—particularly when compared to the wide availability of data we have today. In addition to this scarcity, it is reasonable to assume that data accuracy was also lower compared with our current capacity to collect and consolidate data for subsequent analysis.

I would sincerely like to see a more consistent justification for choosing this specific outbreak. I did not find (or at least not in sufficient detail) in the article which factors led the authors to apply this proposal to the 1857 yellow fever outbreak in Lisbon, rather than to another similar event. It would be important for these justifications to be explained more thoroughly in the text.

**Response to Reviewer #3, Summary and General Comments – A:**  Please refer to the response to ‘Reviewer #3, Editorial and Data Presentation Modifications – G’, where we describe the changes made to the manuscript to strengthen the justification for choosing this specific epidemic.

**(B)** An additional suggestion would be to consult the book "A história da Febre Amarela no Brasil", authored by Dr. Odair Franco (available at: <https://bvsms.saude.gov.br/bvs/publicacoes/0110historia_febre.pdf>), which presents data on historical outbreaks in Brazil and other countries, including a description of the measures adopted to contain the first recorded outbreak in Brazil, in the city of Recife. These elements could enrich the historical review presented in the manuscript.

**Response to Reviewer #3, Summary and General Comments – B:** Thank you for the suggestion. The work by Dr Odair Franco was very interesting; we have now utilised some of the information from this book to help bolster the historical review of YF, with particular consideration of Portugal-Brazil relationships. Franco’s work has specifically helped strengthen the Introduction section where we discuss historical YF and its links to Portugal and Brazil.

References to Franco are included in the following passage, on page 4, lines 109-123:

“YF reached South America in the seventeenth century, with one of the first Brazilian outbreaks occurring in Recife in 1685, imported on a cargo ship from the island of São Tomé (in the Gulf of Guinea, west of Africa) (Franco, 1969). Repeated epidemics in Brazil at this time led to the first health campaign in the ‘New World’ being implemented in 1691; this included the cleaning of houses and streets through bonfires and fumigation, burials of disease victims distant from populated places, establishment of ‘port health police’ who would inspect ships and crews, and confinement of slaves and some women (Franco, 1969). Despite this, YF still became established in Brazil. Franco (1969) describes one ship that had five YF victims on the journey between Recife and Portugal in 1691, indicating the long relationship between Portugal, Brazil, and YF. Portugal-Brazil migration and trade intensified in the 1840s (Chalhoub, 1993), contributing to the ignition of YF outbreaks in Lisbon by mobilising vectors from endemic regions of South America (Esteves, 2014). The importation of YF from Brazil to Portugal was formally recognised by the King of Portugal for causing the 1856 Porto outbreak; Brazil also drove YF to Montevidéo, Uruguay, and Buenos Aires, Argentina in 1857 (Franco, 1969), illustrating the reach of YF from Brazil.”

**(C)** I had the impression that the manuscript did not sufficiently address the potential for generating new cases in places of congregation, such as churches. The analysis seems biased toward the hypothesis that wind was the main — or one of the main — explanations for the spread of the virus by transporting infected mosquitoes. The contributions of human movement during viremia and the role of mild or asymptomatic infections seem underexplored, and I believe these aspects deserve greater attention. If the vector within the household (or very close to it) finds all the conditions necessary for its maintenance, why would mosquitoes seek other locations? Thus, it does not seem necessary for urban mosquitoes to move in search of more favorable environments. If we then imagine a “passive” transport mechanism such as wind, how would this operate indoors?

**Response to Reviewer #3, Summary and General Comments – C:** Thank you for comments. Regarding places of congregation, please refer to ‘Response to Reviewer #3, Editorial and Data Presentation Modifications – J’. Regarding the (passive) movement of vectors, please refer to ‘Response to Reviewer #3, Editorial and Data Presentation Modifications – I’.

**(D)** Similarly, I would like to see more comments on earlier outbreaks and their potential to generate immune individuals who would have been “protected” during the 1857 circulation of yellow fever. The text cites the arrival of yellow fever brought by the RMS Tamar in March 1857, but this seems insufficiently explored, or at least deserving of more detail.

**Response to Reviewer #3, Summary and General Comments – D:** Please refer to the response to ‘Response to Reviewer #3, Editorial and Data Presentation Modifications – F’ regarding our thoughts on earlier outbreaks generating immune individuals. In short, while this is possible, the relatively small scale of the previous outbreaks would suggest that the impact of immune individuals was not considerable.

**(E)** I did not review the abstracts; please make sure to adjust them as needed according to modifications resulting from suggestions or comments.

**Response to Reviewer #3, Summary and General Comments – E:** Thank you. We have ensured to adjust the abstract in line with all reviewer suggestions and comments.

**(F)** When stating that the outbreak was triggered by the arrival of a ship from Belém, Brazil, I would like to see data on the occurrence of yellow fever in that city at the time (if available). There may be information on this in A história da Febre Amarela no Brasil, authored by Dr. Odair Franco (available at the link above) (I’m not sure that it contains that type of information).

**Response to Reviewer #3, Summary and General Comments – F:** We agree that this would be an interesting perspective to add, however, we have not found any data available specific to Belém for the specific years in questions.

Using Dr Odair Franco’s book, we have added some context on YF in Belém to the Results section. Page 9, lines 329-332: “According to reports, YF settled in Belém from 1850 for at least half a century, suffering 5,205 fatalities in this time, and YF spread from here to other Brazilian ports, such as Soure and Vigia (Franco, 1969), which demonstrates the infection potential from the city.”

**(G)** Please review the entire text to correct the use of the abbreviations YF and YFV. On numerous occasions throughout the manuscript, the authors refer to outbreaks/cases of YFV, when the correct term would be outbreaks/cases of YF.

**Response to Reviewer #3, Summary and General Comments – G:**

Please refer to the response to ‘Reviewer #3, Editorial and Data Presentation Modifications – D’, where we describe the changes made to the manuscript to reflect YFV and YF appropriately.

**Reviewer #4:** I consider this study to be highly original,and the manuscript is very well written.The description of the methods,incluinding the study area, the variable collected, and the application of the inference model is particularyly clear and well presented. In the Discussion section, the authors could be more direct in explaining how the data obtained in the study coul be useful for advancing the understanging of yellow fever.

**Response to Reviewer #4, Summary and General Comments:** Thank you for your comments. Please refer to ‘Response to Reviewer #4, Conclusions – A’, where we discuss how this study is useful for understanding YF transmission in present day.

Further, we have added a paragraph on the role of places of congregation on transmission, and how contributing to limited literature on this topic can inform present day understanding and vector control strategies. Please see ‘Response to Reviewer #3, Editorial and Data Presentation Modifications - J‘ for more details.

We also emphasise the significance of our contribution of R_0_ calculations in a pre-vaccination era. This is particularly important given that vaccination against YFV is not common in Europe due to a lack of recent outbreaks, which is an important contribution due to the potential for YF return to Europe.

**Reviewer #5:** This study reviewed and analysed historical records to recreate the spatial dispersal of YF during the 1857 epidemics in Lisbon Portugal. The study also investigated how environmental and sociodemographic factors of the population to this spread. The authors impressively explained their methodology, provided adequate analysis and highlighted limitations in their methodology and ensuing results.

**Response to Reviewer #5, Summary and General Comments:** Thank you.
